# High prevalence group testing in epidemiology with geometrically inspired algorithms

**DOI:** 10.1038/s41598-023-45639-6

**Published:** 2023-11-02

**Authors:** Hannes Schenk, Yasemin Caf, Ludwig Knabl, Christoph Mayerhofer, Wolfgang Rauch

**Affiliations:** 1 Unit of Environmental Engineering, University of Innsbruck, Technikerstraße 13, 6020 Innsbruck, Austria; 2 Tyrolpath Obrist Brunhuber GmbH, Hauptplatz 4, 6511 Zams, Austria

**Keywords:** Infectious diseases, Public health, Epidemiology, Population screening, Laboratory techniques and procedures

## Abstract

Demand for mass surveillance during peak times of the SARS-CoV-2 pandemic caused high workload for clinical laboratories. Efficient and cost conserving testing designs by means of group testing can substantially reduce resources during possible future emergency situations. The novel hypercube algorithm proposed by Mutesa et al*.* 2021 published in *Nature* provides methodological proof of concept and points out the applicability to epidemiological testing. In this work, the algorithm is explored and expanded for settings with high group prevalence. Numerical studies investigate the limits of the adapted hypercube methodology, allowing to optimize pooling designs for specific requirements (i.e. number of samples and group prevalence). Hyperparameter optimization is performed to maximize test-reduction. Standard deviation is examined to investigate resilience and precision. Moreover, empirical validation was performed by elaborately pooling SARS-CoV-2 virus samples according to numerically optimized pooling designs. Laboratory experiments with SARS-CoV-2 sample groups, ranging from 50 to 200 items, characterized by group prevalence up to 10%, are successfully processed and analysed. Test-reductions from 50 to 72.5% were achieved in the experimental setups when compared to individual testing. Higher theoretical test-reduction is possible, depending on the number of samples and the group prevalence, indicated by simulation results.

## Introduction

Managing the SARS-CoV-2 pandemic required containment and mitigation strategies, such as mobility restrictions, as well as public health and social interventions. Extensive individual testing was undertaken by many countries. Among the most extensive testing countries were Austria, Cyprus, Denmark and UAE with over 10^4^ tests per a thousand people since the start of the pandemic^1^. Epidemiological testing of individuals requires great effort in terms of organization, resources and logistics. The most vulnerable people in lower developed countries with less resources for mass testing are likely impacted the most by the pandemic^2^. With limited surveillance capacity it is important to establish an efficient, accurate and sustainable diagnostics pipeline^3^. Experience of the SARS-CoV-2 pandemic has shown the importance of rapid diagnostics infrastructure, which may eventually also be beneficial to future emergencies or situations of urgent mass testing^4^. Diagnostics laboratories have the task to cope with mass testing endeavours in pandemic emergencies, which can pose a challenge in terms of workload.

A possible solution for managing large quantities of tests is to pool samples together and perform tests on the grouped samples^5^. If all of the samples in a group are negative, the grouped test is negative. If one or more of the samples in a group is positive, the grouped test is positive. The scientific field of group testing investigates sparse recovery problems, that identify algorithms and test designs to efficiently detect defective items (for example positive SARS-CoV-2 samples) in a large group. This statistical and combinatorial approach allows to reduce testing equipment and processing time by applying an appropriate pooling design. The pooling design is a set of instructions on how to effectively pool samples, in order to maximize the probability of fully identifying all defective items with a minimum of testing effort^6^. Prerequisite for successful group testing is a sufficiently small number of defective items (i.e. low group prevalence) and an appropriate number of samples within one group test, to not violate the limit of detection^7^.

Group testing is applicable to a wide range of scientific fields, including biology, medicine, computer science and engineering^8^. Dorfman^9^ initiated the field by identifying syphilitic soldiers during Second World War by using a simple pooling design, batching samples without overlap. The main research in recent years is focused on non-adaptive group testing, by exploring approaches to optimize the pooling design^10,11^, as well as improving the decoding properties^12,13^. A recent work by Mutesa et al.^14^ in *Nature* proposed a pooling algorithm by considering the geometric properties of a hypercube.

This paper explores the hypercube algorithm proposed by Mutesa et al.^14^ for the application of SARS-CoV-2 testing, by adaptation to higher prevalence settings (~ 10%). The hypercube design allows to vary geometric parameters, such as number of edge nodes and cube dimension. Thereby, the characteristics of the algorithm are altered, which influences the performance of the pooling design. As part of their proof of concept, Mutesa et al.^14^ propose the use of a hypercube with three edge nodes and adjusting the cube dimension according to the group size. Theirproposed testing design is applied sequentially, until every item is labelled with assertiveness as defective or non-defective. This approach is very effective for low prevalence applications (~ 0–2% defective items), but restricts the group size to be a whole power of three.

In this work, the pooling design is altered and tailored towards higher prevalence, with the aim of maximizing correct labelling after one parallel test round. To clearly rephrase the aim of this study, high prevalence epidemiological group testing with the emphasis on practicality and applicability are the goals of this work. The contributions of the undertaking are implemented with the following tasks. In a numerical study, different performance indicators are inspected to optimize the parameter selection of the pooling design by performing Monte Carlo simulations. Empirical validation is performed on SARS-CoV-2 virus samples with group sizes ranging from 50 to 200, characterized by group prevalence up to 10%. At the end, a practical example is presented, explaining how to apply the proposed methodology.

## Methodology

**Figure 1:**
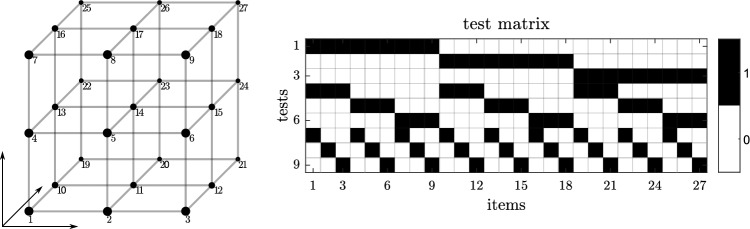
Left: Example cube with *L* = 3 edge nodes in *D* = 3 dimensions. Each node represents an item, tests are formed by slicing the cube perpendicular to the principal directions. Right: Test matrix **M** resulting from the cube design on the left.

Non-adaptive group testing consists of two distinct phases, the design phase and the decoding phase^15^. The test design for non-adaptive group testing is prepared in the design phase. Common practice in the field is to express the test design as a matrix (see Fig. 1 right for an example). A binary testing matrix **M** displays the grouping instructions for *N* items into *t* tests. The columns *j* of **M** represent each item (or SARS-CoV-2 sample), the rows *i* of **M** represent each test. The design matrix has the following properties: Element (*i*, *j*) is 1, if the *i*-th item is included in the *j*-th tests and 0 otherwise. After performing the tests, the binary vector **y** of length *t* is obtained, displaying the test results (1 defective, 0 non-defective). In the decoding phase, the set of defective items is estimated by analysing the vector **y**. For the present application, all items in negative test sets are labelled as non-defective.

The following subsections expound on the Hypercube algorithm, as well the performed numerical, analytical and experimental investigations. At the end of this manuscript, a practical example is carried out on how to apply the proposed methodology. The public GitHub repository https://github.com/HannesSchenk/Adaptive-Hypercube-Group-Testing shares the code that generates design matrices. No human participants are involved in the study. Test results from SARS-CoV-2 samples were provided by an external laboratory. All Methods and experimental undertakings are carried out in accordance to guidelines and regulations.

### Group testing with Hypercube pooling

Hypercube pooling is a group testing algorithm, inspired by the geometrical properties of a hypercube. A hypercube can be characterized by the number of edge nodes *L* and the dimension *D*, with the number of nodes *N* = *L*^*D*^. Figure 1 displays an example hypercube arrangement with *N* = 27 items (left, *L* = 3, *D* = 3) and the corresponding test matrix (right), outlining the grouping instructions. Each test consists of items, corresponding to the slices perpendicular to each principal direction *D*. The subgroups formed therefrom constitute the pooling design. The example test matrix in Fig. 1 shows the pooling instructions matching the cube lattice in the figure. For example, test number 3 contains the items 19 to 27, derived from a planar slice of the cube.

A hypercube with *L*^*D*^ items is partitioned into *LD* overlapping subgroups, consisting of *L*^(*D−*1)^ items each. This grouping arrangement allows to efficiently detect one or few defective items. It works extremely well for low prevalence (~ 0–2% of items are defective) and can confirm to identify one defective item, when the condition *D* = sum(**y)** is met, without further retesting. However, for higher prevalence setups, naive hypercube pooling layouts risk to have few (or no) negative tests, requiring extensive retesting of suspects and low to no reduction of resources. Therefore, refining the properties of hypercube pooling by altering the geometric characteristics is proposed.

For applicability to epidemiological SARS-CoV-2 testing, the aim is to maximize the information output after one round of testing. After the first round of parallel group tests, a subset of items is identified as suspects *N*_sus_. For conformation of infection, the suspect items need to be retested individually. If a correct test design is chosen for a given group of items, the subset of suspects is much smaller than *N*. The efficient nature of the pooling strategy allows to reduce test resources considering all required tests (including grouped tests and retesting of suspects).

### Adapted hypercube pooling

A numerical study is conducted to evaluate the optimal set of hypercube parameters for a given group of items *N*, of which *k* are defective. The construction of an efficient test design requires a prevalence assumption (i.e. estimate the number of defective items). With the application of SARS-CoV-2 testing, laboratories have good knowledge about prevalence with regard to different sources of samples (i.e. hospitals, public institutions, voluntary samples etc). Without a prevalence assumption, a conservative test design is required, potentially resulting in lower pooling performance.

The layout of the hypercube can adapt to any combination of *L* and *D*, with $\{L,D\}\in {\mathbb{N}}$. This provides flexibility to satisfy a range of applications with varying number of items and group prevalence. Adapting the hypercube algorithm to higher prevalence is achieved by introducing “dummy items”, which are non-defective per definition. Dummy items fill out the hypercube lattice in case of *L*^*D*^ > *N*. Choosing higher values for *L* and *D* than required for a given pool of *N* samples, inflates the cube lattice with non-defective dummy items and thereby lowers the group prevalence artificially. This approach creates a sparse distribution of defective items on the hypercube lattice and artificially lowers the prevalence, at the cost of having to perform more tests. Note, dummy items need not to be processed in the testing phase and are merely a conceptual tool for the optimal selection of *L* and *D*. In order to avoid unequal cardinality of tests, we propose to distribute dummy items uniformly over the cube lattice.

The aim of the algorithm is to efficiently identify *k* defectives out of *N* items. In a random virtual setting with a given cube design, the placement of *k* defective items on the geometric grid influences the performance of the testing design. Depending on the random placement of—for example − 5 defectives on the lattice, different test reductions are achieved. Evidently, defective items in a real-world scenario are randomly allocated to a node. In order to examine the varying pooling performance influenced by the random placement of defectives over the lattice, a numerical study performing Monte Carlo simulations is carried out. In principle, the sample space of possible configurations of defective item distribution over the hypercube lattice can be computationally brute forced, however the computational effort for sensible setups is exceedingly large. Moreover, the Monte Carlo approach is particularly applicable in this case, since the entire sample space maps to few distinct solutions (i.e. same number of positive tests), all having equal pooling performance each.

In order to attain the optimal hypercube parameters for a varying set of items and group prevalence, a numerical study is carried out on a space of configurations. Systematically varying the parameters *L*, *N* and *k*, while performing Monte Carlo simulations within each configuration, randomly placing *k* defectives on the hypercube lattice, allows to examine the group testing performance for a range of hypercube test matrixes and prevalence setups. For the numerical study, groups with up to 750 items with a group prevalence up to 18% are analysed.

**Figure 2:**
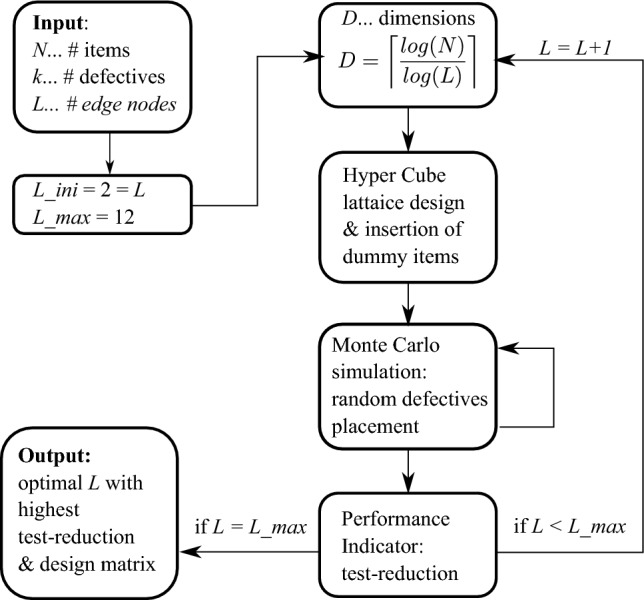
Flowchart outlining the algorithmic procedure and hyperparameter optimization of the numerical study.

Figure 2 displays a flowchart, outlining the procedure of the numerical study in detail. For any particular set of *N*, *L* and *k*, the process in the flowchart is followed. The process starts with initializing the parameters *N*, *k* and *L*. The hypercube dimension is than computed by ceiling the fraction of the logarithms of *N* and *L*. From this, the number of nodes is determined by *L*^*D*^. The surplus of nodes over *N* items is compensated with dummy items. The dummy items are distributed uniformly over the lattice, preventing large disparities in the size of pooled tests. For this given setting of lattice design, Monte Carlo simulations randomly distribute defective nodes. This allows to examine the average test-reduction as well as the standard deviation therefrom. The standard deviation illustrates the susceptibility to deviate from the mean. The lower the standard deviation, the greater the probability of achieving the predicted test-reduction by the test design. By looping through a predetermined set of edge nodes (in the numerical study from *L* = {2…12}), an optimum of test-reduction can be determined, corresponding to a specific design matrix.

Optimal parameters can be selected based on various performance indicators, such as test-reduction, resource consumption, number of suspects *N*_sus_, failure probability, among others. Practically, the parameter selection for the optimal test design is to be tuned with consideration of the respective application. Using a combination of performance indicators, it is possible to express a cost function that aligns with a particular application and enables informed decision-making regarding the optimal model parameters for a given case.

### Limitations of adapted hypercube pooling

Hypercube pooling is designed to label items as defective or non-defective correctly and efficiently. Assuming noise free testing (no false positive or false negative outcome of test results), in theory, hypercube pooling guarantees to label all defective items as suspects. Only non-defectives can potentially be mislabelled. This is a result of the decoding protocol, labelling only items in negative tests as non-defective.

To fully understand the characteristics of the pooling approach, the following edge cases can be analytically considered. Depending on the random placement of defectives on the cube lattice, a best-case and a worst-case scenario can be outlined. The best-case scenario occurs, if all defectives are placed in a single test, resulting in the identification of all defectives, without mislabelling any non-defectives as suspects. This is guaranteed to occur in the trivial case of *k* = 1, regardless of the pooling design. This instance is recognised by exactly *D* tests being defective. *D* tests is also the minimum number of defective tests, as long as at least one item is defective. The possibility for the occurrence of the best-case scenario theoretically exists, as long as there are less or equal defectives than the number of items in a test (i.e. *k* ≤ *L*^(*D−*1)^). Although improbable, but by chance, all defectives could be located in one single test, allowing to label all non-defectives correctly.

On the other hand, the worst-case occurs, when by chance the defective items lie on the diagonal of the cube lattice (or form a Latin hypercube formation, that can be rearranged into a diagonal with simple row/column order manipulation^16^). Therefrom follows, that each defective item pollutes *D* tests. Considering this worst-case and *k* ≥ *L*, no negative test (or *t* defective tests) occur in **y**, resulting in *N* = *N*_sus_. The result is a failure of the group test in the sense that no test-reduction is achieved. In case of *k* < *L* and—by chance—all defectives pollute *D* tests, *D*(*L *− *k*) tests are guaranteed to be negative, guaranteeing to have some test-reduction even in the worst-case scenario. The probability of all defectives lying on the diagonal for the case of *k* ≤ *L* can be calculated with

1 $$p=\frac{{\prod }_{a=0}^{k-1}{\left(L-a\right)}^{D}}{k!\left(\genfrac{}{}{0pt}{}{{L}^{D}}{k}\right)}.$$

In other words, *p* is the probability, that each of the *k* defectives pollutes *D* tests. The expression divides favourable outcomes by possible outcomes. The numerator counts the outcomes that satisfy the condition of polluting *D* new tests. The binomial coefficient in the denominator expresses all possibilities to place *k* defectives on the cube lattice, multiplied by the permutation factor *k*! to compensate for unordered sampling without replacement.

### Validation experiments

In order to empirically validate the claims of the group testing algorithm, three pooling designs are tested on SARS-CoV-2 samples in a laboratory setting. Previously confirmed positive samples were pooled with negative samples according to selected test designs, validating the methodology. Table 1 lists the details of the three performed experiments.

**Table 1:** Validation experiments.

Experiment	Group characteristics			Pooling design		
	*N*	*k*	Prevalence (%)	*L*	*D*	Estimate mean test-reduction (%)
I	50	5	10	5	3	34.9
II	100	5	5	10	2	62.7
III	200	5	2.5	7	3	72.8

*N*… total items; *k*… defective items; *L*… edge nodes; *D*… dimension.

As described in Table 1, three experimental group tests are performed with 50, 100 and 200 samples respectively and 5 defective items each. The hypercube characteristics of the tests are listed, as well as the mean test-reduction (*TR*), estimated from Monte Carlo simulations, varying the hypercube parameters, performing 10^4^ simulations each. Estimated test-reduction is calculated as percentage from the individual test requirement *N* and considers all required tests, comprising the tests from the pooling design (*LD*), as well as retesting *N*_sus_ suspects. It is computed with

2 $$TR=\frac{\left(LD\right)+{N}_{sus}}{N} 100\mathrm{\%}.$$

The hypercube settings for the empirical validations are selected based on two criteria. Firstly, the test-reduction was considered as a selection metric. Secondly, pooling designs with lower number of groups are preferred, which reduces the manual grouping effort of the laboratory. The design matrices **M** of the pooling designs used for the experiments are attached in the Supplementary Material.

Samples were pooled in Eppendorf tubes as described above and underwent RNA extraction using the chemagic^®^ Viral DNA/RNA 300 Kit in a chemagic 360 instrument for nucleic acid extraction (PerkinElmer, Massachusetts, USA) according the manufacturer’s instructions. SARS-CoV-2 was detected by using the ViroReal^®^ Kit SARS-CoV-2 & SARS (Ingenetix GmbH, Vienna, Austria) according to instructions for use. The limit of detection for this kit is 21 copies per PCR reaction. The Ct-values of the positive samples ranged from 20 to 30.

### State-of-the-art and contributions

The Sars-CoV-2 pandemic gave rise to a multitude of group testing publications in recent years. In this subsection we shortly explore the state-of-the-art literature concerning epidemiological group testing. Furthermore, we aim to explain the contributions of this work to the field at hand.

In the first year of the pandemic Hogan et al.^17^ and Abdalhamid et al.^18^ performed group tests for Sars-CoV-2 surveillance. They lead the way in providing a proof of concept for the application of group testing for SARS-CoV-2 as an effective strategy to conserve testing resources. The benefits of non-adaptive group testing approaches concerning practicality and time criticality are recognized by Price and Scarlett^10^ and McDermott et al.^19^. Price and Scarlett^10^ propose a probabilistic group testing algorithm based on the binary splitting approach. McDermott et al.^19^ use combinatorial test strategies on varying prevalence settings which outperform Dorfman sequential approaches. Täufer^20^ explores Shifted Transversal pooling designs and rigorously derives error probabilities for multipool setups. Test-efficiency (required tests per item) and optimal pool size are investigated by Hanel and Thurner^21^. In their study, a formula for optimal pool size is presented, as well as a practical example is carried out, displaying the test efficiency if the population of Austria were to be tested. Regen et al.^22^ demonstrate analytical derivations for an optimal pool size and give a practical guideline on how to apply a two-staged pooling strategy for laboratories. An innovative approach called Polynomial Pools introduced by Brust and Brust^23^ constructs pooling matrices based on projective geometries and guarantees to correctly label all items up to a specified number.

In this paper, the hypercube algorithm introduced by Mutesa et al.^14^ is adapted and applied to high prevalence settings. Abundant literature in the field investigates group testing with low prevalence settings (see Refs.^14,20,22,23^). Low prevalence testing facilitates high test-reduction by a variety of group testing algorithms. Exploring the high prevalence range in epidemiological group testing is underrepresented in the literature. With this paper we aim to contribute to this research gap and to provide a practical guidance on how to apply efficient one-stage test designs.

## Results

The adapted hypercube pooling method presents a promising tool for epidemiological group testing. Adapting geometric parameters of the cube lattice provides extensive flexibility and allows to tackle a great range of pooling problems. This work investigates optimal test designs for SARS-CoV-2 sample testing, however the results are applicable to a variety of pathogens in epidemiological surveillance. The results section is divided into two parts, firstly the results of the numerical study is presented. Secondly, the experimental validation is covered.

### Numerical results

A group testing problem consisting of *N* items—among which *k* are defective—can be tackled with the hypercube algorithm by varying the geometric properties *L* and *D*. For a given case of *N* items and edge nodes *L*, the dimension *D* results from the following relation

3 $$\mathrm{D}=\left\lceil\frac{\mathrm{log}\left(N\right)}{\mathrm{log}\left(L\right)}\right\rceil,$$

where the brackets ⌈ ⌉ denote the *ceil* function, rounding to the next greater integer. Remaining nodes in the cube lattice are filled with dummy nodes. The number of dummy nodes is given by *L*^*D*^−*N*. For sets of *N*, *L* and *k*, Monte Carlo simulations are performed, evaluating the efficiency of the pooling design. Simulations were performed by systematically altering the parameters *N*, ranging from 5 to 750 in increments of 5, *L* ranging from 2 to 12 in increments of 1, and the prevalence ranging from 0.2% to 18% in increments of 0.2. These configurations resulted in 148,500 individual pool testing setups, for which 10^2^ Monte Carlo simulation have been performed each. The Monte Carlo approach is utilized in the numerical study to account for the possibilities of random distribution of defective items over the hypercube lattice. Varying pooling performing occurs depending on the random allocation of defectives. The influence of the random distribution of defectives is examined by the standard deviation from the mean test-reduction. Furthermore, a larger parameter space (i.e. *N* > 750) can be solved for, however the conducted numerical study aims for epidemiological applications, where larger sample sizes are unsuitable.

**Figure 3:**
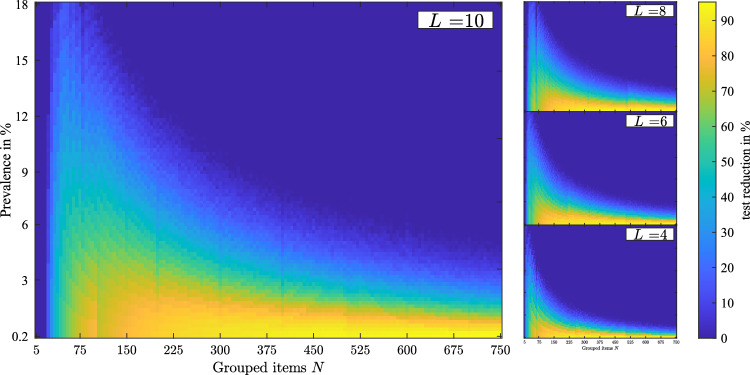
Monte Carlo results outlining test-reduction over *N* and the group prevalence in % for the cases *L* = {10, 8, 6, 4}.

Figure 3 displays the test-reduction in percentage, referenced to the test requirement for individual tests. Test-reduction considers the tests required for the pooling design, as well as the tests to confirm or reject defectivity of suspects. For the simulation setup visualized in Fig. 3, *k* is estimated by the prevalence and *N* by rounding to the nearest integer. Additionally, the constraint *k* ≥ 1 is applied. Test-reduction is depicted as colour profile, where bright shaded areas correspond to high and dark shaded areas correspond to low test-reduction. For SARS-CoV-2 sample testing, relative test-reduction in reference to individual testing is the objective of the optimization, however an arbitrary cost function is feasible in accordance with the respective application.

Numerical results show that high test-reduction (80% and above) is achievable in case of low prevalence and high number of items. Lower number of items allow for higher prevalence to achieve good test-reduction (see hyperbolic outline in Fig. 3). Depending on the number of edge nodes *L*, group testing below a certain threshold of samples is not beneficial. In case of *L* = 10, *N* below 30 does not yield test-reduction, in case of *L* = 6, *N* below 20 does not yield test-reduction. This threshold correlates positively with *L* and approximately follows 2*L* + 5. Overall, high prevalence decreases the performance of pooling due to the increase of positive tested groups. The more positive groups occur, the less samples can be labelled as negative in the decoding phase, while incidentally labelling negative samples as suspects.

**Figure 4:**
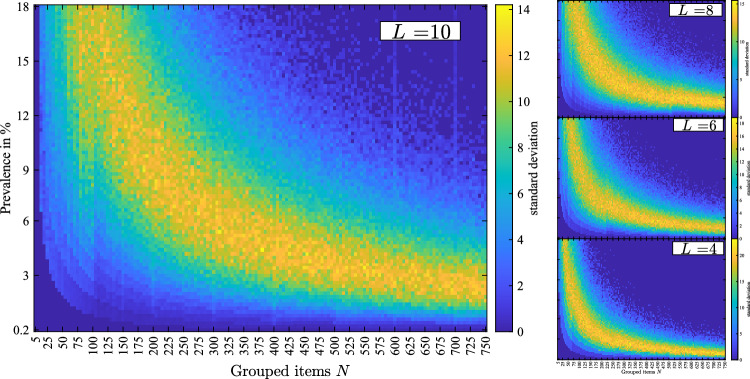
Monte Carlo results outlining the standard deviation from the mean test-reduction.

To examine the resilience of the methodology, the standard deviation from the mean test-reduction is computed. In conformity with Fig. 3, the following figure shows the standard deviation from the mean test-reduction. In general, we can observe that systematically more deviation from the mean is present in a transition area between low prevalence/low number of grouped items and high prevalence/high grouped items. On the bottom left of the figure, approximately 0% standard deviation is observed, outlining the ease of converging to a robust solution. On the top right of Fig. 4, low standard deviation is observed. In these high prevalence cases group testing is not beneficial and all solutions converge to 0% test-reduction. The lower the standard deviation, the higher the precision of the proposed design matrix to perform the predicted test-reduction.

Hypercube pooling performance is compared to Dorfman pooling. Dorfman test designs are simple non-overlapping batches of test groups. In this numerical investigation, the group size of the Dorfman pools is constructed to be $\lceil\sqrt{\mathrm{N}}\rceil$, in order to adapt to a wide range of number of samples. Within the domain examined for the numerical study (*L* = {3…12}*, N* = {5…750} and prevalence = {0.2…18%}, on average 12.5% higher test-reduction is observed with hypercube pooling as compared to Dorfman pooling.

### Experimental validation

Three pooling configurations are empirically validated in experiments. Experiment I with 50, II with 100 and III with 200 SARS-CoV-2 samples have been pooled, containing 5 confirmed positive samples each. This resulted in 10%, 5% and 2.5% group prevalence respectively. The test designs have been selected to maximize test-reduction against individual tests, determined by numerical optimization. The full design matrices for the empirical validation is added to the Supplementary Material.

**Figure 5:**
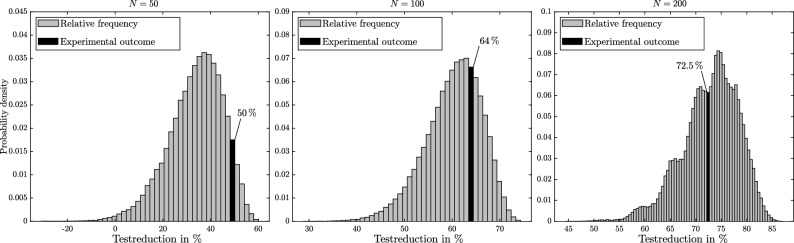
Test-reduction relative frequency numerical results of hypercube pooling of the three experimental setups. The black bars show the measured result.

The measured test-reduction of the three experiments is 50%, 64% and 72.5%, corresponding to the experiments I, II and III respectively. Figure 5 shows the normalized relative frequency of expected outcomes of each experiment, numerically evaluated by Monte Carlo simulations (10^5^ each). The measured test-reductions are highlighted as black bars. In the experiments I and II, slightly above mode performance can be observed, while experiment III turned out marginally lower than the estimated mode value.

## Conclusion

Group testing applied to epidemiology allows to effectively reduce costs and processing time by testing pooled samples, compared to individual testing. Elaborately pooling selected samples into subgroups allows to efficiently identify infected samples out of a large group. The hypercube algorithm proposed by Mutesa et al.^14^ promises high test-reduction for cases with low group prevalence. In this work we adapt the hypercube algorithm towards higher group prevalence. Alteration of key geometrical parameters allow to tune the pooling design for specific needs of the application. Pooling designs are numerically optimized and empirically validated.

Experimental validation shows agreement to the proposed test designs optimized by numerical simulation. Experiments with 50, 100 and 200 SARS-CoV-2 samples have been processed with 10%, 5% and 2.5% group prevalence respectively. Compared to individual testing, up to 72.5% of test-reduction was achieved. Numerical simulation results suggest that higher test-reduction is possible depending on the case specificities. A wide range of settings have been numerically investigated with Monte Carlo simulations (up to 750 samples and 18% group prevalence). Simulation results have been compared to the conventional Dorfman pooling, which is most frequently applied in laboratory practice, if group testing is applied. The adapted hypercube pooling design displays significant increase in pooling performance (12.5% more test-reduction on average). An analytic expression for the failure probability is shown, allowing to adapt the pooling design accordingly. The selection of an optimal pooling design requires a prevalence estimation. Laboratories have usually good knowledge about group prevalence based on the source of samples (i.e. hospitals, public institutions, voluntary samples etc.). Group testing designs can be tailored for different settings and the optimization cost function tuned for different applications.

The proposed testing design by adapting the hypercube algorithm displays advantages in terms of applicability towards higher prevalence settings and temporal criticality. The methodology is designed to maximize the information output after one round of group testing. Group testing as a field of applied mathematics provides solutions to a wide range of practical applications beyond epidemiological surveillance. An efficient and resource conservative approach to mass testing is vital to detect infected individuals in a population.

## Practical example

In the following, a practical example of the proposed methodology is applied. The prerequisite for the application of adapted hypercube pooling is stating two parameters. Firstly, the number of items *N* (or test samples) to be tested. Secondly, a prevalence estimation is required to optimally construct the design matrix for a given setting. The prevalence estimation *k* is stated as an absolute value approximating the number of defective items (or infected samples) from the items to be tested. The output of applying the proposed methodology is the design matrix, showing the optimal grouping of the items into test according to the adapted hypercube algorithm.

The algorithm determines the optimal hyperparameters of the hypercube, subject to maximizing the test-reduction. The proposed algorithm can be computed as a MATLAB code, available on the public GitHub repository https://github.com/HannesSchenk/Adaptive-Hypercube-Group-Testing. The first code section requires the inputs *N* and *k*.
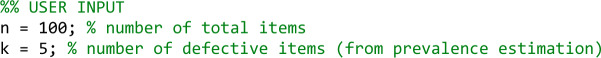


**Figure 6:**
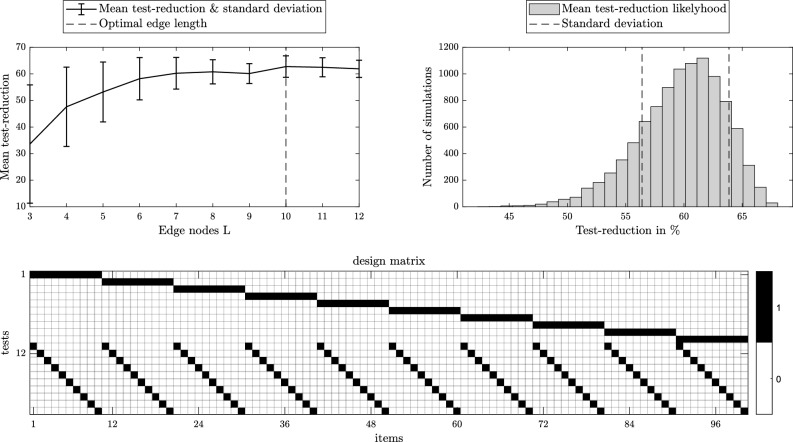
Execution summary of the code on the public GitHub repository with *N* = 100 and *k* = 5.

After running the code, a figure summarising the results of the specific setup is displayed (Fig. 6 shows an example). Furthermore, a csv-file is created, generating the design matrix. Columns of the design matrix represent the items/samples and the rows represent the individual pool tests to be carried out. On the top left, the figure shows the development of test-reduction over the edge length *L* and its optimal value (highlighted by a vertical line). Secondly, it shows the probability distribution histogram, outlining the most likely test-reduction to occur (evaluated from Monte Carlo simulations), in addition to the standard deviation. On the bottom of the figure, the proposed design matrix is displayed graphically. The ‘Design_Matrix.csv’ file is generated and consists of 0 and 1 elements outlining the grouping scheme.

### Supplementary Information

Supplementary Information 1.

Supplementary Information 2.

Supplementary Information 3.

## Data Availability

The datasets used and analysed during the current study are available from the corresponding author on reasonable request.
